# Analysis of the Effect of Increased α2,3-Sialylation on RTK Activation in MKN45 Gastric Cancer Spheroids Treated with Crizotinib

**DOI:** 10.3390/ijms21030722

**Published:** 2020-01-22

**Authors:** Meritxell Balmaña, Francisca Diniz, Tália Feijão, Cristina C. Barrias, Stefan Mereiter, Celso A. Reis

**Affiliations:** 1 i3S—Instituto de Investigação e Inovação em Saúde, Universidade do Porto, 4200-135 Porto, Portugal; 2 IPATIMUP—Institute of Molecular Pathology and Immunology, University of Porto, 4200-135 Porto, Portugal; 3 Institute of Biomedical Sciences of Abel Salazar—ICBAS, University of Porto, 4050-313 Porto, Portugal; 4 INEB—Instituto de Engenharia Biomédica, University of Porto, 4200-135 Porto, Portugal; 5 Medical Faculty, University of Porto, 4200-319 Porto, Portugal

**Keywords:** 3D cell culture, crizotinib, gastric cancer, glycosylation, MET, receptor tyrosine kinase, RON, sialylation, spheroids, tyrosine kinase inhibitor

## Abstract

In the scenario of personalized medicine, targeted therapies are currently the focus of cancer drug development. These drugs can block the growth and spread of tumor cells by interfering with key molecules involved in malignancy, such as receptor tyrosine kinases (RTKs). MET and Recepteur d’Origine Nantais (RON), which are RTKs frequently overactivated in gastric cancer, are glycoprotein receptors whose activation have been shown to be modulated by the cellular glycosylation. In this work, we address the role of sialylation in gastric cancer therapy using an innovative 3D high-throughput cell culture methodology that mimics better the in vivo tumor features. We evaluate the response to targeted treatment of glycoengineered gastric cancer cell models overexpressing the sialyltransferases ST3GAL4 or ST3GAL6 by subjecting 3D spheroids to the tyrosine kinase inhibitor crizotinib. We show here that 3D spheroids of ST3GAL4 or ST3GAL6 overexpressing MKN45 gastric cancer cells are less affected by the inhibitor. In addition, we disclose a potential compensatory pathway via activation of the Insulin Receptor upon crizotinib treatment. Our results suggest that cell sialylation, in addition of being involved in tumor progression, could play a critical role in the response to tyrosine kinase inhibitors in gastric cancer.

## 1. Introduction

Gastric cancer (GC) is the fifth most common cancer and the third leading cause of cancer related deaths worldwide [1]. GC persists in being a clinically challenging disease due to its usual late diagnosis and high molecular heterogeneity. Despite the progress in the last decades in the cancer therapy field, there are still no effective treatments for GC nor reliable biomarkers for the prediction of therapy response or patient outcome [2].

In the scenario of personalized medicine, targeted therapies are currently the focus of cancer drug development. One strategy of the personalized therapy is the inhibition of activated signaling pathways, either by interfering directly with receptor activation or by interrupting the downstream signaling cascade [3]. These inhibitory drugs can block the growth and spread of tumor cells by interfering with key molecules involved in malignancy, such as receptor tyrosine kinase (RTKs). The activation of RTKs is frequently altered in GC, and around 37% of patients may be potentially treatable with RTK/RAS-directed therapies [4,5]. Among these RTKs, the hepatocyte growth factor receptor (HGFR), also known as MET, has been widely studied to be involved in the oncogenesis of a large number of tumors [6]. The comprehensive molecular evaluation of gastric adenocarcinomas performed within The Cancer Genome Atlas (TCGA) project, resulted in the molecular classification of GC into four subtypes. Tumors showing marked aneuploidy and focal amplification of RTKs, including for example, exon skipping in MET, were defined as tumors with chromosomal instability [7]. The MET receptor signaling pathway is frequently overactivated in gastric cancer [6,8]. Another relevant RTK is the Recepteur d’Origine Nantais (RON) (also known as macrophage-stimulating protein receptor (MSPR) or MST1R) which commonly shows aberrant activation in various tumors due to overexpression and generation of oncogenic variants [9,10]. Alterations in the cellular glycosylation has been shown to modulate the activation of MET and RON in GC [11,12,13].

Glycosylation is a complex and highly regulated process of the cell that plays important roles in many cellular events, including cell-cell adhesion and communication, cell-matrix interaction, and response to microenvironmental cues. Aberrant glycosylation has a major impact on the acquisition of malignant features in the tumor progression [14,15,16]. The increase of sialic acid moieties in the cell surface is a shared characteristic of many tumors [17,18,19]. Sialic acids can be added to glycoconjugates by one of at least 20 different sialyltransferases that have different acceptor specificities [20,21].

Currently several therapeutic agents targeting MET and RON are tested in clinical and preclinical phases [9,22,23]. Crizotinib, commercialized as Xalkori^®^, is an oral receptor tyrosine kinase inhibitor (TKI) targeting MET, RON, and ALK [24] that was approved by the FDA for the treatment of non-small cell lung cancer in 2011. It is also currently in clinical trials for other tumors, including GC (NCT02435108, NCT03620643 and NCT02465060). Despite the great expectations placed on these targeted treatments, many patients do not respond or become resistant after treatment by mechanisms that are still not fully understood. Therefore, the assessment of the efficacy of novel treatments as well as their mechanisms of resistance must be performed using in vitro models that better simulate the in vivo conditions. In this regard, the 2D setups, which have been classically used for the culture of mammalian cells, have proven to result in limited prediction accuracy. Noteworthy, 3D cell culture systems can better mimic the complex structures and the physiological conditions of the in vivo tumor [25,26,27,28]. Multicellular tumor spheroids (MCTS), where cells form unique tridimensional complex entities, have been demonstrated to be promising for the screening of novel drugs [29,30,31].

In this work, we have evaluated the role of increased α2,3-sialylation in gastric cancer therapy using an innovative 3D high-throughput cell culture methodology on a MET amplified GC cell line. Our results show that 3D spheroids of MKN45 overexpressing the sialyltransferases ST3GAL4 and ST3GAL6 are less sensitive to crizotinib treatment.

## 2. Results

### 2.1. Overexpression of α2,3-Sialyltransferases Results in Different Cellular Glycocalyx

**Figure 1:**
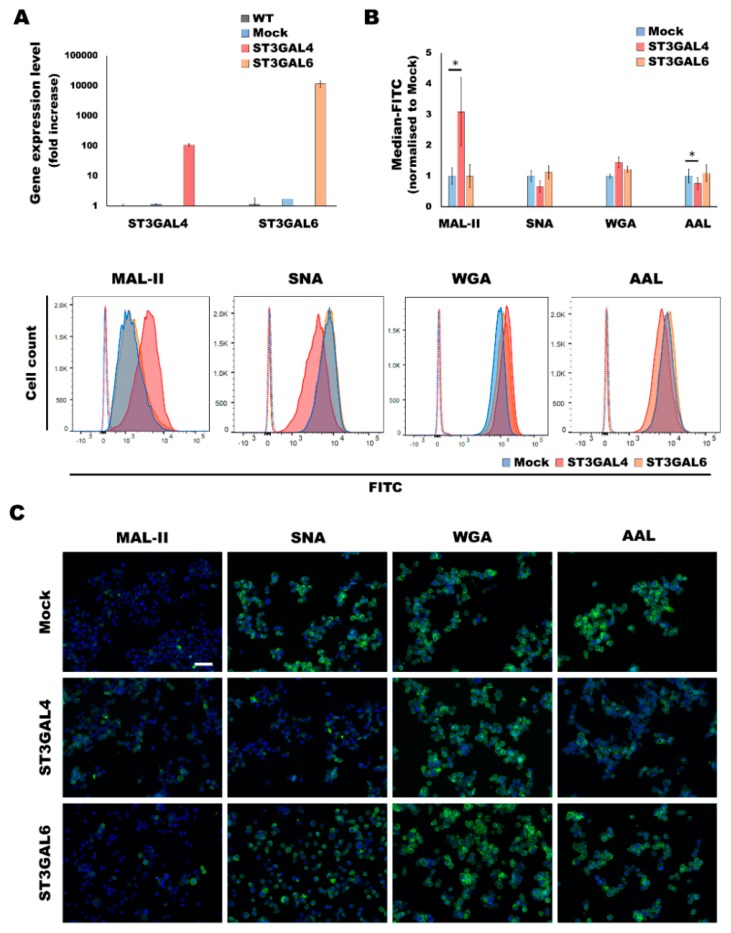
Characterization of the α2,3-overexpressing cancer cell models. (**A**) Expression analysis of the ST3GAL4 and ST3GAL6 mRNA levels of the glycoengineered cell lines and the corresponding control cell lines, wild type (WT) and mock (containing the empty vector) by qRT-PCR. Data represents the average ±SD of 3 technical replicates. (**B**) Flow cytometry analysis of a panel of lectins in MKN45 glycoengineered cell lines as compared to the mock cell line. For each lectin, at least 2 independent experiments were performed. A representative plot for each lectin is also depicted. The negative controls are shown in dotted lines. Statistical significance was determined by student’s *t*-test (*p*-value < 0.05 was considered significant, indicated as *). (**C**) Lectin fluorescent staining of a panel of lectins for sialylation and fucosylation characterization of the gastric cancer cell lines. The cytometry graphs and the immunofluorescence images are representative results of at least 2 independent replicates. Scale bar represents 50 μm.

In a previous work, the human gastric cancer cell line MKN45, which showed low or undetectable expression levels of the sialyltransferases ST3GAL4 and ST3GAL6, respectively, was stably transfected with the expression vector containing the respective full length cDNA encoding for ST3GAL4 and ST3GAL6 [32]. We have determined the expression of the sialyltransferases ST3GAL4 and ST3GAL6 by RT-PCR after normalization with the 18S ribosomal RNA. The ST3GAL4 and ST3GAL6 clones showed an increased expression of approximately 100-fold and 10,000-fold, respectively, compared to the mock (Figure 1A). The ST3GAL6 increase could be considered a de novo expression, since its expression in both wild type and mock is negligible.

The overexpression of the α2,3-sialyltransferases ST3GAL4 and ST3GAL6 led to the expression of a different glycosylation profile in the cell line (Figure 1B,C). The panel of lectins used to characterize the glycocalyx of cells that overexpress ST3GAL4 or ST3GAL6 consisted of Maackia amurensis lectin II (MAL-II), which recognizes sialic acid with α2,3-linkage and Sambucus nigra agglutinin (SNA) which preferentially binds to α2,6-sialic acid moieties. Additionally, we have also addressed the reactivity of the lectin Wheat germ agglutinin (WGA), which primarily binds to β1,4-GlcNAc-linked residues but also has been reported to show binding to heavily α2,3-sialylated glycoproteins [33].

Our results showed that the ST3GAL4 cell model displays a significant increase of α2,3-sialylation concomitant to a decrease of α2,6-sialylation. These results are in agreement with glycomic data previously reported [12,34]. In addition, a significant decrease in fucosylation, presumably outer arm fucosylation, was found as detected by Aleuria aurantia lectin (AAL). Regarding the ST3GAL6 model, we could observe a tendency of increased α2,3-sialylation and fucosylation (Figure 1B,C).

### 2.2. Overexpression of α2,3-Sialyltransferases Affects the Response to Crizotinib

**Figure 2:**
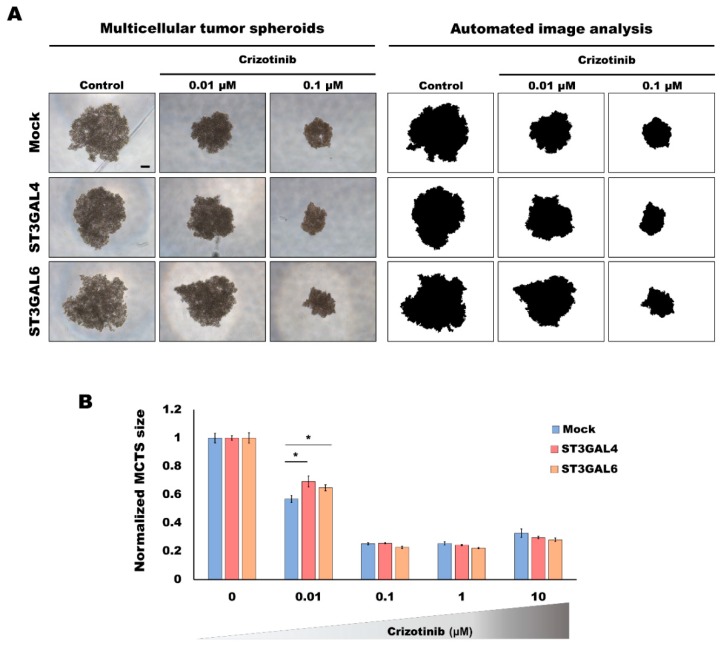
Analysis of the response to tyrosine kinase inhibitor (TKI) treatment of the α2,3-overexpressing cancer cell models grown in 3D. Response to crizotinib treatment evaluated by automated image analysis of gastric multicellular tumor spheroids (MCTS) generated using ultra-low attachment (ULA) 96-well round-bottomed plates for 5 days and treated with different concentrations of crizotinib for 48 h. (**A**) Representative examples of the automated image analysis performed with the open-source Fiji software to determine the size of the gastric MCTS from images acquired with a Leica DMi1 microscope. Scale bar represents 200 μm. (**B**) Graph represents the size variation in each spheroid after 48 h of treatment. Values are means ± SD of at least *n* = 3 spheroids. For each condition, 2 independent experiments were performed. Statistical significance was determined by student’s *t*-test (*p*-value < 0.05 was considered significant, indicated as *).

To investigate whether the different glycosylation displayed by our cell lines had an effect on the capacity of cells to respond to cancer drugs, we evaluated the GC cell models under 3D culture systems and subjected these gastric MCTS to the TKI crizotinib, a drug described to target the RTKs MET and RON. In order to analyze the viability of gastric MCTS when subjected to a range of different concentrations of the inhibitor, an automated image system was used [30] (Figure 2A). The capacity of the different glycoengineered cell line models to grow as 3D spheroids was affected by TKI treatment, and gastric MKN45 MCTS shrank with increasing concentrations of the inhibitor (Figure 2). This dose effect assay revealed a plateau effect at concentrations ≥0.1 µM (Figure 2B). To assess the viability of the spheroids when subjected to crizotinib, we evaluated the proliferative status of the gastric MCTS. Both the western blot and immunofluorescence indicated a markedly reduction of the proliferation at doses higher than 0.1 µM crizotinib (Supplementary Material Figure S1A). Considering the intermediate concentration of 0.01 μM, in which cells were still viable, we could observe significant differences among the cell line models, with cells overexpressing ST3GAL4 or ST3GAL6 displaying higher resistance to the treatment (Figure 2B).

### 2.3. Analysis of the Activation of MET and RON by Overexpression of α2,3-Sialyltransferases

A previous work with the PHA-665752 TKI suggested an increased MET activation as a potential mechanism for resistance in the ST3GAL4 overexpressing model [11]. To investigate whether this was the underlying mechanism of the increased tolerance to the TKI in our 3D model system, we performed western blot and immunofluorescence analyses of the RTK activated forms, pMET and pRON. Considering the results described in the previous section, the evaluation of the RTK activation was performed using the intermediate inhibitory concentrations of 0.05 and 0.1 μM of crizotinib as well as the complete inhibition (1 μM).

**Figure 3:**
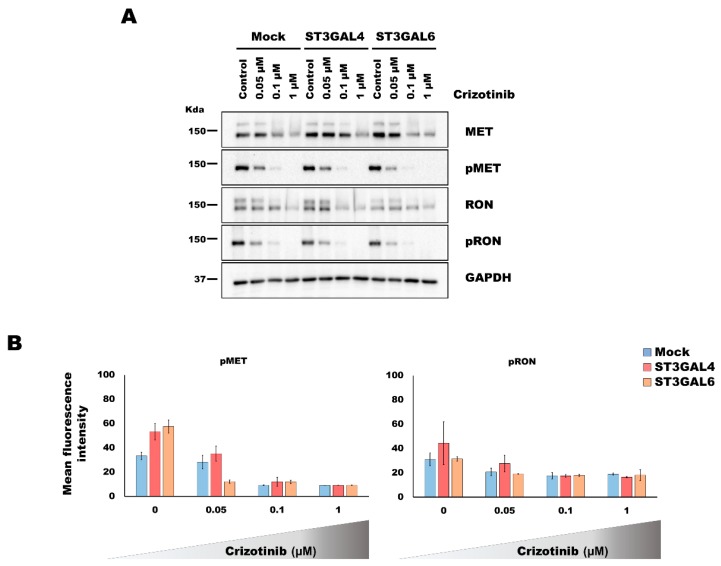
Evaluation of the activation of the tyrosine kinase receptors MET and Recepteur d’Origine Nantais (RON) in MKN45 gastric multicellular tumor spheroids (MCTS) overexpressing α2,3-sialylation after crizotinib treatment. Gastric MCTS were generated using the 3D Petri Dish^®^ technology (MICROTISSUES^®^) for 5 days and treated with different concentrations of crizotinib for 48 h. (**A**) Western blot analysis of MET and RON receptor tyrosine kinases and their activated forms, pMET and pRON. GAPDH was used as a loading control. (**B**) Results of the automated image analysis of the immunofluorescence staining of gastric MCTS (Supplementary Material Figure S1) subjected to different concentrations of crizotinb. No significant differences were found (Student’s *t*-test, *p*-value > 0.05).

The analysis of RTK activation by western blot did not show an increased activation in the MCTS of the glycoengineered cell models (Figure 3A). Although slight differences became evident in immunofluorescence analysis of pMET and pRON in cells overexpressing ST3GAL4 or ST3GAL6, when compared to the control cell line (Supplementary Material Figure S1B,C), the automated quantification of the fluorescent staining disclose no significant differences (Figure 3B).

Concomitant to the decrease of the RTK activation, we could observe a reduction of the RTK protein expression, especially of the immature form (corresponding to the higher molecular weight band). We cannot exclude that the treatment and the consequent decreased activation of cellular signaling could alter gene expression and stability of RTK expression levels.

The results obtained with crizotinib were further validated with a different MET inhibitor, PHA-665752, displaying the same tendencies (Supplementary Material Figure S2).

### 2.4. Overexpression of α2,3-Sialyltransferases Promotes Survival through the Activation of Insulin Receptors

**Figure 4:**
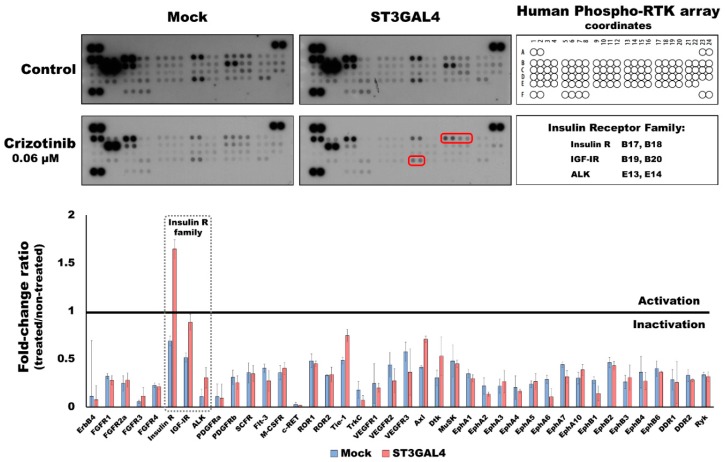
Insulin receptor is activated in ST3GAL4 cell model after treatment with crizotinib. Gastric MCTS were generated using the 3D Petri Dish^®^ technology (MICROTISSUES^®^) plates for 5 days and treated with 0.06 μM of crizotinib for 48 h. 300 μg of protein lysate of mock and ST3GAL4 were subjected to phopho-receptor tyrosine kinases (RTK) arrays. The optical density signal was quantified only in non-saturated spots and measured in duplicates. Upper-left corner spots were not quantified due to overlapping signal. Insulin receptor family members are framed in red and dashed squares.

Cancer cells are complex entities that can modulate their activity under stress conditions and thereby bypass the targets of inhibitory molecules. To monitor the changes in RTK activation upon TKI treatment, we evaluated these targets applying a phospho-RTK array. The results showed that most RTKs were less activated upon treatment. This is in accordance with the crosstalk between the members of the RTK superfamily [35]. However, in cells overexpressing ST3GAL4, the Insulin receptor (IR) showed a 1.7-fold increase after treatment (whereas mock 0.7-fold change) (Figure 4). Remarkably, the 3 members of the insulin family (IR, IGF-IR, and ALK) showed to be increasingly activated in the ST3GAL4 model, when compared to the mock treated with crizotinib, suggesting the activation of insulin family RTKs as a compensatory mechanism, not observed in the control cell line.

## 3. Discussion

Despite important advances in the cancer therapy, the prognosis for patients that present GC in advanced stages remains poor. In many patients, targeted therapeutics fail due to unknown intrinsic or acquired resistance mechanisms of tumor cells. In this regard, the understanding of the mechanisms of response to treatment will provide new tools for patient stratification.

In non-malignant cells, the activation of RTKs and downstream signaling pathways is reversible and tightly regulated, rendering cells dependent on extracellular cues from the environment. However, in cancer cells these pathways are often constitutively activated [36]. Molecular therapies targeting specific RTKs responsible for cell growth, proliferation, survival and migration, have been showing promise as strategies to treat cancer [37].

Despite the encouraging in vitro results with TKIs, there are patients who have the appropriate molecular features that fail to respond due to intrinsic resistance, and almost all patients who initially respond to these therapies eventually develop acquired resistance. In the case of patients undergoing crizotinib therapy, acquired resistance usually occurs within one year after the first administration [24]. Although some of the bypass mechanisms that cells exploit to become resistant to RTK-targeted therapy have been described [38,39], there are still many tumors that are resistant due to unknown mechanisms. The glycosylation machinery of cancer cells is altered leading to diverse aberrant glycosylation profiles of tumor cells and constituting a potential mechanism for the observed differential efficacy of targeted therapy. In this work, we have used glycoengineered GC cell lines displaying MET amplification to address the role of differential cell sialylation in response to crizotinib.

Aberrant glycosylation on the cell surface is considered a hallmark of cancer with potential applications in the clinical setting [16]. In this regard, GC has been shown to express simple *O*-glycan antigens [19], and recently an association between the expression of the glycan antigen Thomsen-Friedenreich (TF) and the MSI status of GC tumors has been described, highlighting this glycan antigen as a biomarker with potential consequences for patient stratification [40].

Previous work disclosed that MET and RON downstream signaling is more activated upon overexpression of ST3GAL4 [11,12]. The expression of the SLeX epitope was detected by antibody-based assays, however, a detailed glycomic analysis by MS made clear that the most striking alteration in ST3GAL4 model was the general increase of α2,3-sialylation [34]. Increased sialylation on RTKs have been shown to play a role in tumor progression [11,41]. Moreover, this altered glycosylation has been proven to lead to different response to monoclonal antibodies or TKIs. It has been recently shown that increased cell sialylation induced lower response of GC cells to the anti-ErbB2 monocolonal antibody trastuzumab [42] as well as reduced response of ovarian cancer cells to the EGFR TKI gefitinib [43]. Considering the high molecular heterogeneity of GC, we can speculate that a minor proportion of cancer cells displaying a differential sialylation could bypass the initial therapy and proliferate, giving rise to a non-sensitive tumor and ultimately leading to clinical resistance, collectively, reinforcing sialylation as a mechanism of cancer therapy resistance. In agreement with this, the analysis of the sialylation status of a resistant and a sensitive to gefitinib lung cancer cell line resulted in an overall sialylation increase of the resistant cells [44].

Crizotinib, a TKI for which we have shown promising in vitro results for the treatment of GC presenting MET amplification/overactivation, is already in clinical setting for the treatment of EML4-ALK–translocated (ALK-positive) non-small cell lung cancer (NSCLC) patients. In GC, 5% of patients present overactivation of the MET receptor and therefore, could benefit from crizotinib treatment, as also shown by others [45,46]. Our results show that increased α2,3-sialylation leads to reduced sensitivity to the TKI, including increased resistance to ALK inhibition. The slight differences observed by the two different models could be attributed to the fact that ST3GAL6 is known to be involved in sialylation of glycolipids rather than glycoproteins [47]. Glycolipids, such as gangliosides, have been shown to be capable of altering RTK activation [14,48]. Nevertheless, both glycolipids and glycoproteins are main components of the cell glycocalyx [15] and, thus, the overexpression of the sialyltransferases ST3GAL4 and ST3GAL6 may play a role in the remodeling of the extracellular matrix of gastric MCTS. It is known that the activation of RTKs can be modulated via interaction with sialylated glycosphingolipids or other glycoconjugates, such as glycosaminoglycans or proteoglycans, present in the extracellular milieu [49,50,51]. Moreover, growth factors, including Hepatocyte growth factor (HGF, the ligand for MET activation), are tethered to the cell surface by interaction with glycoconjugates, facilitating ligand-mediated receptor activation and downstream signal transduction [52,53].

One of the classical mechanism to acquire resistance to RTK inhibition is the activation of an alternative RTK that restores the signaling pathways [38]. In the present work, we have performed an evaluation of the phospho-RTK proteome and disclosed the activation of the IR in the ST3GAL4 overexpressing cells in response to the inhibition of the MET and RON RTKs using an innovative 3D-approach. Moreover, previous glycoproteomic analysis showed that IR displayed increased sialylation on *N*-glycan site N445 in the ST3GAL4 overexpressing cell model in comparison to the mock cells [34]. We cannot clearly implicate a direct crosstalk between MET and IR, however, we hypothesize that other intermediaries or indirect crosstalk may be important as has been shown with intracellular Src kinase [54]. On the other hand, MET has been implicated as a mediator of resistance to therapies targeting members of the ErbB-RTK family in breast, lung, colon, and GC cells [55,56,57,58,59]. Considering that several studies reveal a particular weakness in strategies targeting single receptors, the future of the targeted therapy field points towards a “rationally-designed cocktail” of targeted drugs and the development of inhibitors targeting multiple RTK or common downstream signaling proteins [60,61]. Moreover, combination therapy could be used to tackle both acquired and intrinsic resistance. Furthermore, the use of molecules targeting *N*-glycosylation, such as the small molecule NGI-1, have shown promising results as a second-hit for cancer cells addicted to RTKs activation and resistant to TKIs [62]. Further development of 3D-cell culture techniques, especially the automation and additional advances of the high-throughput technologies, will certainly contribute to the progress in the drug discovery field.

Overall, our results highlight the relevance of sialylation in cancer therapy response variability. Importantly, it is the first time to the best of our knowledge that the role of glycosylation in GC therapy response has been addressed using 3D-culture. Our system mimics better the tumor features, resulting in an improved model for in vitro drug screening. Moreover, these results lay the basis for future studies using cell glycosylation as a biomarker for prediction of therapeutic response in cancer.

## 4. Materials and Methods

### 4.1. Cell Culture

The gastric carcinoma cell line MKN45 was obtained from the Japanese Cancer Research Bank (Tsukuba, Japan) and was stably transfected with the full length human ST3GAL4 and ST3Gal6 genes and the corresponding empty vector pcDNA3.1 (mock) as previously shown [32]. The cells were grown in monolayer culture in uncoated cell culture flasks. Cells were maintained at 37 °C in an atmosphere of 5% CO_2_, in RPMI 1640 GlutaMAX medium (Gibco, Thermo Fisher Scientific, Waltham, MA, USA), supplemented with 10% fetal bovine serum (FBS, Biowest, Riverside, MO, USA) and in the presence of 0.5 mg/mL G418 (Invitrogen, Waltham, MA, USA). Cultured cell lines were routinely tested for mycoplasma contamination by PCR amplification for mycoplasma pulmonis UABCTIP, mycoplasma penetrans HF-2, and mycoplasma synoviae 53.

### 4.2. RNA Extraction, cDNA Synthesis, and Real-Time Reverse Transcription PCR (RT-PCR)

Total cellular RNA was extracted using the TRIzol Reagent (Invitrogen, Carlsbad, CA, USA) according to manufacturer’s instructions. RNA concentration and purity were determined by NanoDrop ND 1000 Spectrophotometer (NanoDrop Technologies Inc, Wilmington, DE, USA). The reverse transcription was performed with 3 μg of RNA, using random primers and the SuperScriptR IV Reverse Transcriptase Kit (Invitrogen) following the manufacturer’s instructions. RT-PCR was performed with 2 μL of diluted cDNA, 10 mM of each primer, 5 μL SYBR^®^ Green Master Mix (1X) (Thermo Fischer Scientific; former Savant, MA, USA) and ultrapure water in a final volume of 10 μL using the ABI 7500 (Applied Biosystems, Foster City, CA, USA). Negative controls without templates were added for each reaction. Human 18S ribosomal RNA (18S rRNA) was used as housekeeping gene to normalized the data. Thermal cycling conditions used were an initial denaturing step at 95° C for 10 min followed by 40 cycles of 15 s at 95 °C and 1 min at 60 °C. Melting curve analyses were performed to ensure the specificities of the amplification reactions. Relative expression values and standard deviation (SD) have been calculated using the ΔΔCT approach, as previously described [63].

The primer sequences used were as follows: forward 5′-cctggtagctttcaaggcaatg-3′, reverse 5′-cctttcgcacccgcttct-3′ (ST3GAL4), forward 5′-cggctgattttagaaagattgctt-3′, reverse 5′-cggctgattttagaaagattgctt-3′ (ST3GAL6), and forward 5′-cgccgctagaggtgaaattc-3′, reverse 5′-cattcttggcaaatgctttcg-3′ (18S rRNA).

### 4.3. Generation of Gastric Multicellular Tumor Spheroids

Gastric multicellular tumor spheroids (MCTS) formation was conducted by two different approaches, either in ULA plates (Corning, New York, NY, USA) or in 3D Petri Dish^®^ (MICROTISSUES^®^ technology, MicroTissues Inc., Sigma-Aldrich, St. Louis, MO, USA) following the protocol described in [30,64]. Briefly, 12-series agarose micro-molds were prepared using 2% agarose in 0.9% of NaCl. Micro-molds were placed in 12-well plates and equilibrated with RPMI 10%FBS before cell seeding. Cell suspensions of 250 cells/microwell of each cell line were seeded in the corresponding micro-mold. A 30 min incubation period was used for cells to settle into the micro-molds and additional medium was finally added to the wells. Spheroids formation both in ULA plates and 3D Petri Dish^®^ was monitored with the inverted microscope for cell culture Leica DMi1 (Leica Microsystems, Wetzlar, Germany).

### 4.4. Spheroid Treatment

The MCTS response to the RTK inhibitors crizotinib and PHA-665257 (both from Sigma-Aldrich) was followed by monitoring the spheroids growth. Aliquots of 250 cells/well were seeded in the ULA plates. After 5 days, when the gastric MCTS were formed, cells were treated with the inhibitors diluted in cell culture medium at concentrations ranging from 0.01 to 10 μM for 48 h. Control conditions containing the corresponding amount of the vehicle (DMSO) were performed. At day 5 and 7 (before and after treatment), microscope images were acquired using a Leica DMi1 microscope.

For immunofluorescence and protein analyses (western blot and array), after 5 days of MCTS formation in molds as described in Section 4.3, cells were treated with the corresponding TKI concentration (as well as the vehicle control) for 48 h.

### 4.5. Image Analysis of Size of the Gastric Multicellular Tumor Spheroids

The gastric MCTS size was examined as previously described in [30] using the ImageJ image analysis software with the Fiji image processing package [65]. Briefly, a picture of each well of the ULA plate containing one spheroid each was taken at the different time points. All images were processed by color deconvolution “H and E” using the green channel, threshold “minimum” was applied and only objects larger than 62,000 pixels with “include holes” were kept. The resulting mask, which contained a single object, was used to define spheroid size. The size difference for each spheroid was analyzed and the results subjected to statistical analysis. Each independent experiment consisted of a ULA plate containing all the tested conditions in at least triplicates. In order to calculate the Normalized MCTS size, we applied the following calculations:

1. Ratio of MCTS for each MCTS (*x*):
  $$\mathrm{Ratio}\;\mathrm{MCTS}\;\left(x\right)=\frac{\mathrm{Area}\;\mathrm{MCTS}\;\left(x\right)\mathrm{after}\;48h\;\mathrm{of}\;\mathrm{treatment}}{\mathrm{Area}\;\mathrm{MCTS}\;\left(x\right)\;\mathrm{before}\;\mathrm{treatment}}$$
2. Normalized ratio for each MCTS (*x*):
  $$\mathrm{RaNormalized}\;\mathrm{Ratio}\;\mathrm{MCTS}\left(x\right)=\frac{\mathrm{Ratio}\;\mathrm{MCTS}\;\left(x\right)}{\mathrm{Average}\;\mathrm{Ratio}\;\mathrm{of}\;\mathrm{control}\;\mathrm{condition}\;\left(0µM\right)}$$

### 4.6. Flow Cytometry and Lectin Fluorescent Staining

Cells were detached using Gibco^®^ versene solution (ThermoFisher, Waltham, MA) and stained with previously conjugated lectins with FITC-conjugated streptavidin (Invitrogen) for 20 min at 4 °C. Cells were strained, labeled with propidium iodide and measured using BD FACSCanto™ II (BD Biosciences, San Jose, CA, USA) Three independent experiments were conducted. Data were analyzed using FlowJo (BD Biosciences, San Jose, CA, USA).4.4. The biotinylated lectins used where MAL-II (*Maackia amurensis* lectin II), SNA (*Sambucus nigra* agglutinin), WGA (Wheat germ agglutinin) and AAL (*Aleuria aurantia* lectin), (all from Vector Laboratories, Burlingame, CA, USA).

An aliquot of each sample was placed on microscopy slides and let dry overnight at RT in dark. Nuclei were stained with DAPI and slides were mounted with VectaShield (Vector Laboratories) and imaged with Zeiss Axio Imager Z1 microscope equipped with an AxioCam MR ver.3.0 (Carl Zeiss, Oberkochen, Germany).

### 4.7. Fluorescent Cell Staining of Gastric Spheroids

The gastric MCTS generated with 3D Petri Dish^®^ (as described in Section 4.3) and treated (as described in Section 4.4) with different amounts of crizotinib and PHA-665257 were fixed with 4% *w*/*v* PFA for 30 min at RT. Fixed samples were paraffin-embedded and the blocks were cut into 5 μm sections. Slides were deparaffinized in xylene and rehydrated in sequentially decreasing ethanol concentrations prior to immunofluorescence staining. In brief, slides were blocked with non-immune goat serum in 10% PBS and incubated overnight with the corresponding dilution of the primary anti-human antibody. Slides were then washed with PBS and incubated with the secondary antibody anti-rabbit IgG conjugated with Alexa Fluor^®^-488 for 1 h at RT. Nuclei were counterstained with DAPI and samples were mounted with VectaShield (Vector Laboratories). Sections were imaged with Zeiss Axio Imager Z1 microscope equipped with an AxioCam MR ver.3.0 (Carl Zeiss).

The antibodies used were: p-MET (Tyr1234/1235, D26 clone, Cell Signaling Danvers, MA, USA), and p-RON (Y1238/Y1239, R&D Systems, McKinley Place, MN, USA). These antibodies were used after antigen retrieval with Tris/EDTA pH 9 solution (Novocastra™ Epitope Retrieval Solutions, Leica Biosystems) and diluted 1/100. Ki-67 (ab15580, Abcam, Cambridge, UK, dilution 1/500) used after antigen retrieval with citrate buffer pH 6.

### 4.8. Image Analysis of Receptor Activation

The fluorescent staining from MET and RON activation upon crizotinib treatment of the gastric MCTS described in Section 4.7 was examined using the ImageJ image analysis software with the Fiji image processing package [65]. All images were analyzed by applying the “color threshold” function to subdivide the pixels of the green channel into groups based on their intensity. The pixels of each group were quantified and the result was used to calculate a mean intensity value for each image. At least 2 different images per condition were analyzed.

### 4.9. Western Blot

Total protein lysates were collected from 48-h-treated gastric MCTS (described in Section 4.4) using lysis buffer 17 (R&D Systems, Minneapolis, MN, USA) supplemented with 1 mM sodium orthovanadate (Sigma-Aldrich, St. Louis, MO, USA), 1 mM phenylmethanesulfonyl fluoride (PMSF) (Sigma-Aldrich) and cOmplete^TM^ protease inhibitor cocktail (Roche, Basel, Switzerland; Sigma-Aldrich). Protein concentration of each condition was determined using the DC protein assay (BioRad, Hercules, CA, USA). Same amounts of total protein lysates were electrophoresed under reducing conditions on polyacrylamide gels and transferred onto nitrocellulose membranes (GE Healthcare Life Sciences, Chicago, IL, USA). After 1 h blocking at room temperature, membranes were incubated overnight at 4 °C with primary antibodies. Then, membranes were washed and incubated for 1 h at room temperature with the corresponding peroxidase-conjugated secondary antibodies (Jackson ImmunoResearch, Cambridgeshire, UK). Chemiluminescence signal was obtained using the ECL western blot detection reagent GE Healthcare and visualised with Chemidoc (BioRad)

The antibodies used were: MET (3D4, ThermoFisher Scientific, Waltham, MA, USA, dilution 1/1500), RON (E-3, Santa Cruz Biotechnology, Dallas, TX, USA, dilution 1/100), p-MET (Tyr1234/1235, D26 clone, Cell Signaling Danvers, MA, USA, dilution 1/1500), p-RON (Y1238/Y1239, R&D Systems, McKinley Place, MN, USA, dilution 1/500), GAPDH (0411, Santa Cruz Biotechnology, dilution 1/2000), and Ki-67 (ab15580, abcam, Cambridge, United Kingdom, dilution 1/500).

### 4.10. Phosphoprotein Array

Five-day gastric MCTS were treated with 0.06 μM of crizotinib for 48 h (as described in Section 4.4) and lysed in lysis buffer 17 (R&D Systems) supplemented with 1 mM sodium orthovanadate, 1 mM phenylmethanesulfonylfluoride (PMSF) and protease inhibitor cocktail (Roche, Basel, Switzerland). The protein concentration of the lysates was determined by the DC protein assay (BioRad) and the recommended total protein amounts were used for the human phospho-RTK (R&D Systems). The array protocol and the subsequent analysis of the results were performed according to manufacturer’s instructions. The optical density signal of each spot was quantified using the Image Lab Software (BioRad) under non-saturating conditions. Upper-left corner spots were not quantified due to saturation and overlapping signal. Each RTK is present in duplicate in the array, therefore each bar in the graph represents the average of the signal of the two spots of the treated sample normalized to the control (non-treated). A value equal to 1 means no difference between crizotinib-treated and untreated sample. A value higher or lower than 1 represents either increased activation or inactivation of the RTK upon treatment, respectively.

## Supplementary Materials

Supplementary materials can be found at https://www.mdpi.com/1422-0067/21/3/722/s1.

## Abbreviations

AAL	*Aleuria aurantia* lectin
EGFR	Epidermal growth factor receptor 1
ErbB2	Epidermal growth factor receptor 2
GC	Gastric cancer
HGFR	Hepatocyte growth factor receptor
IR	Insulin receptor
MAL-II	*Maackia amurensis* lectin II
MCTS	Multicellular tumor spheroids
MSI	Microsatellite instable
NSCLC	Non-small cell lung cancer
RON	Recepteur d’Origine Nantais (also known as macrophage-stimulating protein receptor (MSPR) or MST1R)
RT-qPCR	Real-time quantitative PCR
RTK	Receptor tyrosine kinase
SNA	*Sambucus nigra* agglutinin
TF	Thomsen-Friedenreich
TKI	Tyrosine kinase inhibitor
ULA	Ultra-low attachment
WGA	*Wheat germ* agglutinin
