# Supplementary material for: Analysis of the Effect of Increased α2,3-Sialylation on RTK Activation in MKN45 Gastric Cancer Spheroids Treated with Crizotinib
Source: Int J Mol Sci. 2020 Jan 22;21(3):722. doi: 10.3390/ijms21030722 (PMC7037121; doi:10.3390/ijms21030722)
Supplement: Supplementary file 1 [file ijms-21-00722-s001.pdf]

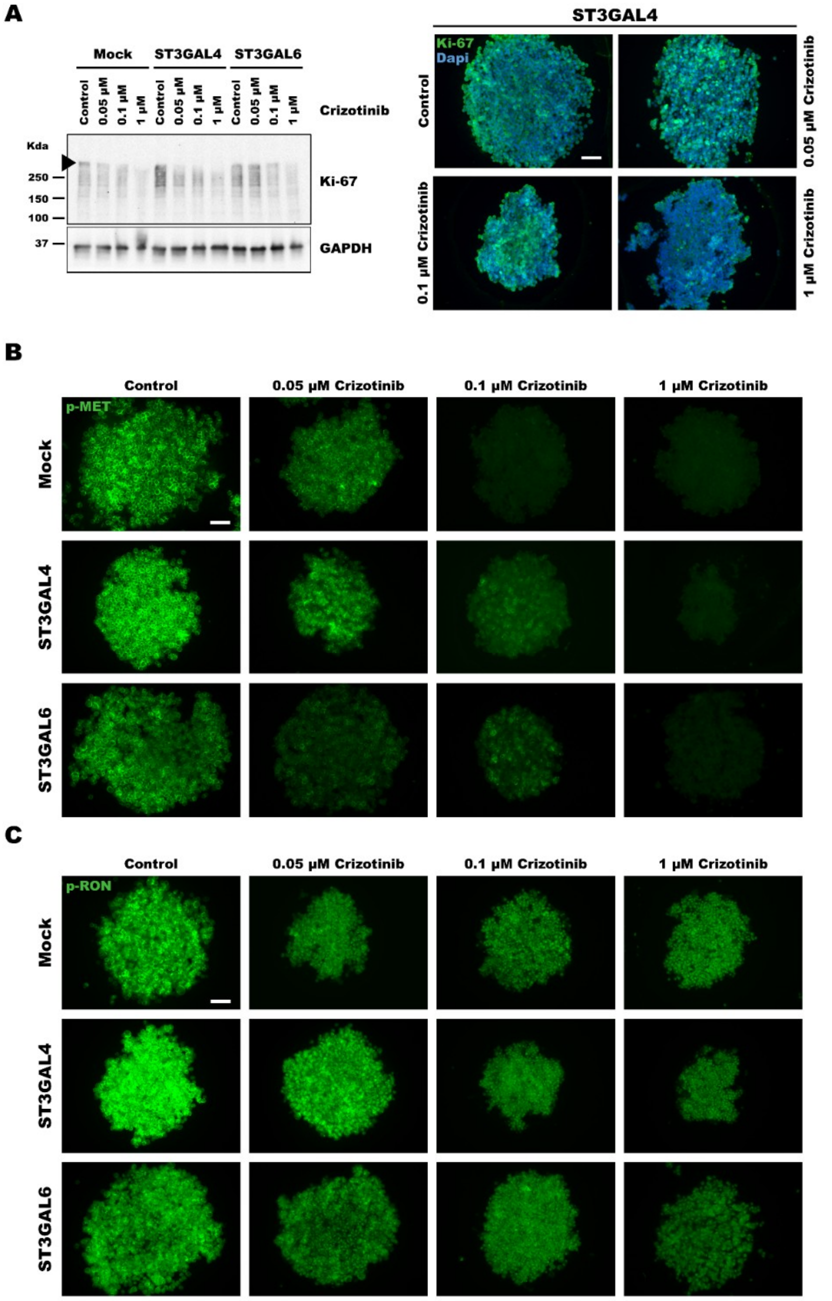

Figure 1. **Analysis of the response to TKI treatment crizotinib of the** α**2,3-overexpressing cancer cell models grown in 3D.** Gastric MCTS were generated using the 3D Petri Dish® technology (MICROTISSUES®) for 5 days and treated with different concentrations of PHA-665257 for 48 h. (**A**) Western blot and immunofluorescent staining of the proliferation marker Ki-67 of gastric MCTS (**B-C**). Immunofluorescent staining of (**B**) pMET and (**C**) pRON of the treated gastric MCTS. Scale bar represents 50 µm.

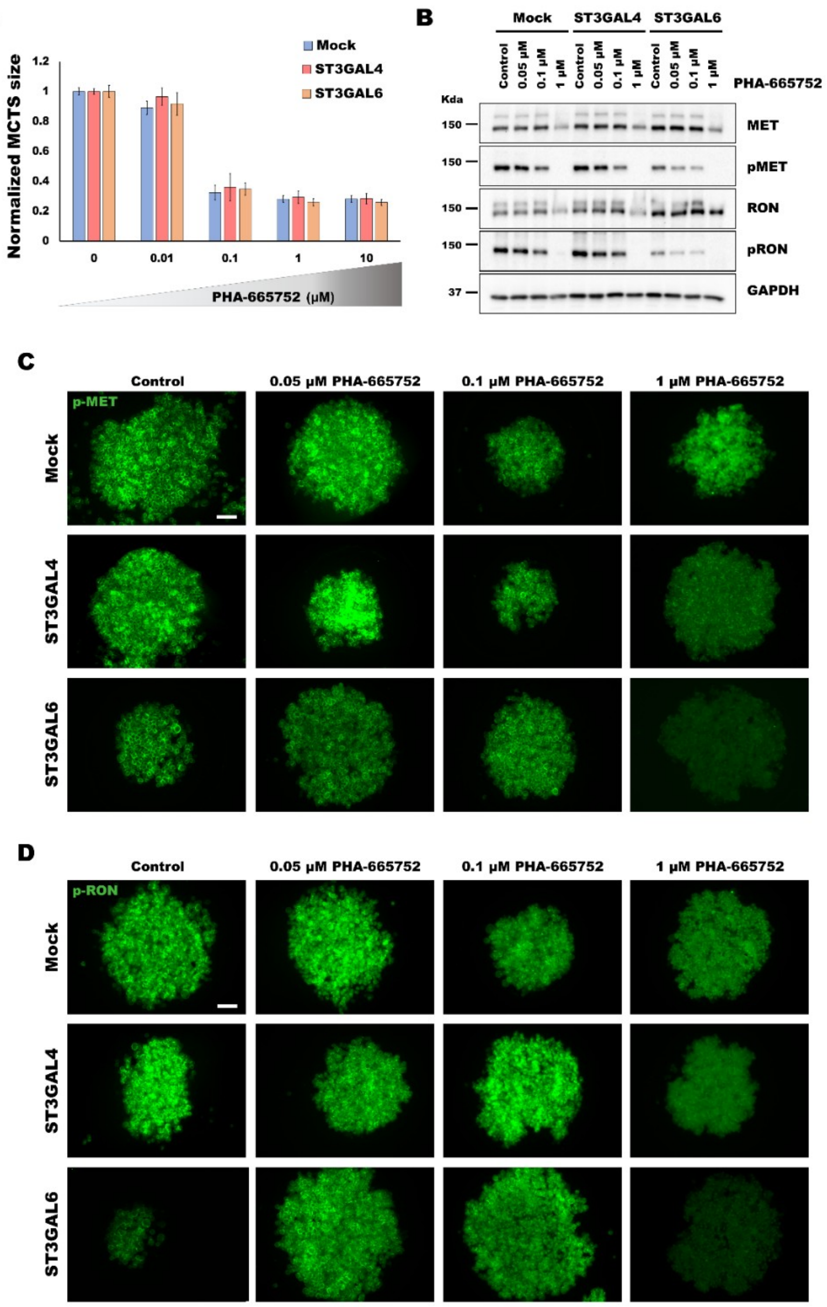

Figure S2. Analysis of the response to TKI PHA-665752 treatment of the α2,3-overexpressing cancer cell models grown in 3D. (**A**) Gastric multicellular tumor spheroids (MCTS) were generated using ultra-low attachment (ULA) 96-well round-bottomed plates for 5 days and treated with different concentrations of PHA-665257 for 48 h. Response to PHA-665752 treatment evaluated by size using automated image analysis. Graph represents the size variation in each spheroid after 48 h of treatment. Values are means ± SD of at least n=3 spheroids. For each condition, 2 independent experiments were performed. (**B-D**) Gastric MCTS were generated using the 3D Petri Dish® technology (MICROTISSUES®) for 5 days and treated with different concentrations of PHA-665257 for 48 h. (**B**) Western blot analysis of MET and RON receptor tyrosine kinase and their activated forms, pMET and pRON. GAPDH was used as a loading control. (**C**) Immunofluorescent staining of pMET and (**D**) pRON of the treated gastric MCTS. Scale bar represents 50 μm.
